# Diagnosis

**Published:** 1866-02

**Authors:** Henry S. Chase


### DIAGNOSIS.

By Henry S. Chase, M.D.,D.D.S.

*Failures* in diagnosis are too common. In a full practice are we not too prone to be *hasty* in our examinations of the teeth. I was particularly impressed with the subject to-day owing to the following

### CASE.

Mr. W. called for advice. He had been to his dentist twice, in about a week after having teeth plugged, to get relieved of the toothache in two teeth that had just been plugged with amalgam. The operator, an excellent dentist, had bled the gums over the affected teeth at the first visit, thinking the trouble, *periostitis*, originating from thermal changes in the pulp, caused by the amalgam plugs. The patient could take no fluids in the mouth at a different temperature from the blood without excruciating pain. The teeth had never ached until plugged.

As bleeding the gums did not relieve the patient, he called upon his dentist again, who, after examining the case, decided that he would have to lose the teeth, and recommended immediate extraction. Not liking the advice the patient left, and came to me.

He pointed out the first and second right superior molars as the ones that ached. I found an amalgam plug of moderate size on the anterior proximal surface of the first molar, and a similar one on the posterior proximal surface of the second molar.

The teeth were in as close approximation as possible. These teeth showed me no signs of periostitis; neither sore on pressure nor loose. At first I thought the pain might proceed from galvanic action between the two plugs. And doubtless it was thus caused, for they had never ached until plugged. I took the most delicate excavator I have, and passed it between the teeth, at their necks, and what was my gratification in finding an immense cavity in each tooth at this point.

Cutting the teeth apart, I found these cavities extending to the pulps. I applied arsenical paste and shall save them.

There were no external signs of decay here; no discoloration.

I wish to take no credit to myself in discovering the trouble in this case; I may have made similar mistakes also. But it was certainly a very great oversight in the practitioner that he did not make an examination *between* these teeth. Every dentist should be provided with an extremely delicate instrument like an excavator for the purpose of finding cavities of decay. It is of especial use, not only *between* teeth, but for probing the *seams* on the grinding surfaces of molars and bicuspids.

Since writing the above, a few days have passed, and I have seen the patient, who has had no pain since the end of the third hour after receiving the arsenical paste; the pulps are removed and the teeth ready for plugs.

